# Supplementary material for: Image quality guided smart rotation improves coverage in microscopy
Source: Nat Commun. 2020 Jan 9;11:150. doi: 10.1038/s41467-019-13821-y (PMC6952408; doi:10.1038/s41467-019-13821-y)
Supplement: Supplementary file 1 — Supplementary Information [file 41467_2019_13821_MOESM1_ESM.pdf]

Supplementary information

Image quality guided smart rotation improves coverage in microscopy

He et.al

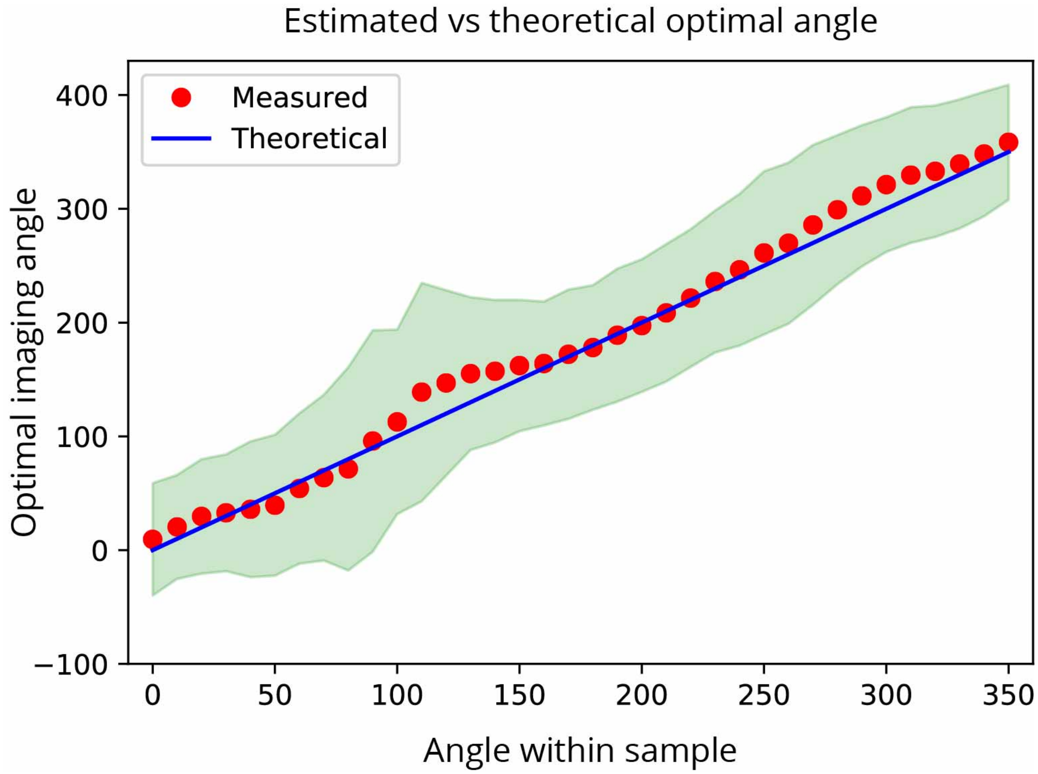

Supplementary figure 1: Optimal imaging angle for angular slices vs the location of the angular slices. Green envelope denotes full width half maximum area of the von Mises distribution.

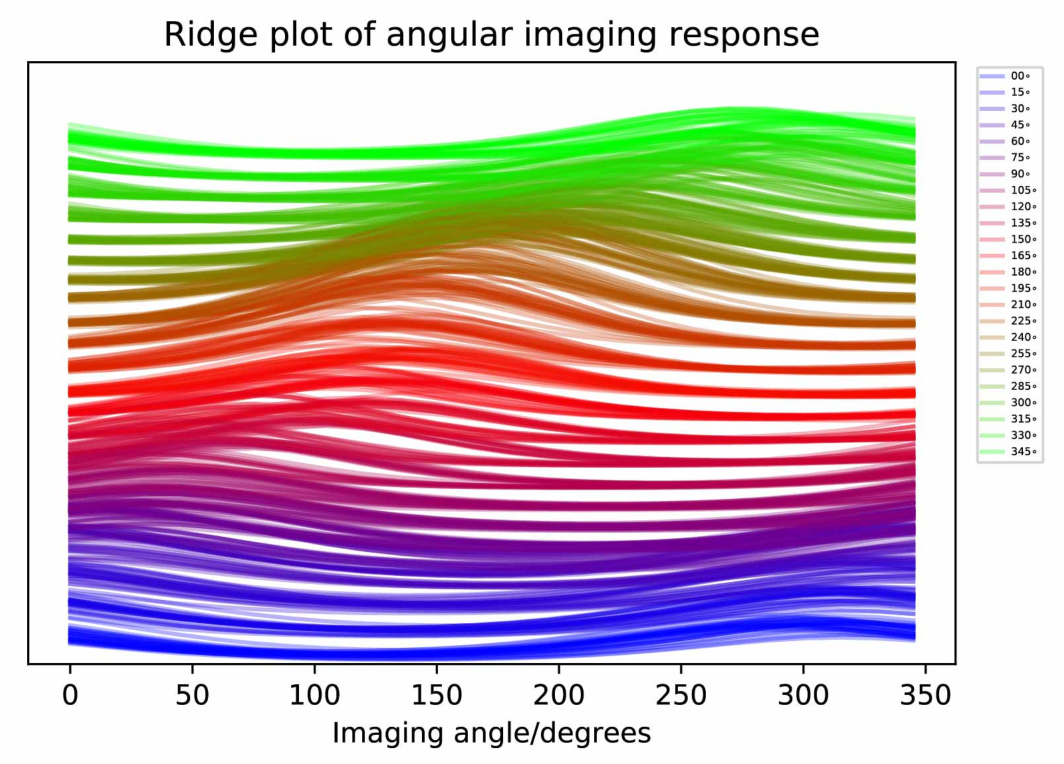

Supplementary figure 2. Ridge plot of imaging response of different angular slices of a zebrafish embryo over time.

Supplementary Note 1

Notes on implementing the workflow into other SPIM systems

Currently, the software on the github repository https://github.com/henryhetired/smartrotationjava can be run as a command line software to evaluate the sample coverage of a multi-view SPIM dataset and return the optimal angle combination given the number of angles needed. During imaging, the software runs on a separate computer to the microscope control computer. The software listens to commands sent by the control computer via TCP/IP to perform the necessary analysis and return with results. The capture machine then needs can parse the results and reconfigure for the subsequent imaging steps. Theoretically, the smart rotation workflow can be integrated into any SPIM where the uses have access to the underlying control software. The detailed command communication structure can be found on the repository. Even if it is not possible to integrate the smart rotation workflow into the image acquisition process, users can acquire a 24 view dataset and run the sample coverage estimation manually. This allows the user to find an optimal configuration at the beginning of the experiment which is often still better than blind angle selection.
