# Supplementary material for: Image quality guided smart rotation improves coverage in microscopy
Source: Nat Commun. 2020 Jan 9;11:150. doi: 10.1038/s41467-019-13821-y (PMC6952408; doi:10.1038/s41467-019-13821-y)
Supplement: Supplementary file 3 — Description of Additional Supplementary Files [file 41467_2019_13821_MOESM3_ESM.pdf]

**Description of Additional Supplementary Files**

**File name:** Supplementary Movie 1

**Description:** Anterior-posterior view of a zebrafish embryo (Tg(h2afva:h2afva-mCherry),48hpf) imaged with 24 different equally spaced angles with an mSPIM. Images are maximum intensity projections along the y axis.

**File name:** Supplementary Movie 2

**Description:** Image response curve of different angular region. Scatter plot is the estimated number of foreground blocks and connected curve is the fitted von Mises distribution curve.

**File name:** Supplementary Movie 3

**Description:** Evolution of ridge plot of the imaging angular imaging response of a zebrafish embryo ( Tg(kdrl:GFP), 48 hpf) over a 16 hour imaging experiment.

**File name:** Supplementary Movie 4

**Description:** Top panel: comparison of 3-view fused image data acquired with blind multi-view and the smart rotation workflow. Scale bar represents 150 µm.

Mid panel: Relative difference in information content between the two approaches.

Bottom panel: Cumulative information content gain over the entire volume by using the smart rotation workflow over the blind approach. Green segments represent information gain, red segments represent information loss.
